# Advance directives as a tool to respect patients’ values and preferences: discussion on the case of Alzheimer’s disease

**DOI:** 10.1186/s12910-018-0249-6

**Published:** 2018-02-20

**Authors:** Corinna Porteri

**Affiliations:** Bioethics Unit, IRCCS Centro San Giovanni di Dio Fatebenefratelli, Via Pilastroni, 4 - 25125 Brescia, Italy

**Keywords:** Alzheimer’s disease, Dementia, Advance directives, Attorney in fact, End of life decisions, Personal identity

## Abstract

**Background:**

The proposal of the new criteria for the diagnosis of Alzheimer’s disease (AD) based on biomarker data is making possible a diagnosis of AD at the mild cognitive impairment (MCI) or predementia/prodromal– stage. Given the present lack of effective treatments for AD, the opportunity for the individuals to personally take relevant decisions and plan for their future before and if cognitive deterioration occurs is one the main advantages of an early diagnosis.

**Main body:**

Advance directives are largely seen as an effective tool for planning medical care in the event the subject becomes incompetent. Nevertheless, their value has been questioned with regard to people with dementia by scholars who refer to the arguments of personal identity and of patient’s changing interests before and after the onset of dementia.

In this paper, I discuss the value of advance directives in Alzheimer’s disease and other kind of dementia. Despite critics, I argue that advance directives are especially advisable in dementia and provide reasons in favor of their promotion at an early stage of the disease as a valuable tool to respect patients’ values and preferences on medical treatment, including participation in research and end of life decisions. I mainly support advance directives that include both decisions regarding health care and the appointment of an attorney in fact.

**Conclusion:**

I conclude that patients with AD at a prodromal or early stage should be offered the opportunity to execute an advance directive, and that not to honor a demented individual’s directive would be an unacceptable form of discrimination towards those patients.

## Background

Alzheimer’s disease (AD) is a neurodegenerative, progressive, highly disabling disease for which effective treatments are still not available. It is estimated that about 50 million people worldwide are currently affected by AD and other kind of dementia and this number will rapidly rise in the global population as an increase in life expectancy brings with it age related diseases [1]. Dementia is one of the major causes of disability and dependency among older people worldwide. It causes great individual and family suffering and it represents an overwhelming burden for the individuals who have dementia, for their caregivers and families, and for the society as a whole [2].

With the publication of the new criteria for the diagnosis of Alzheimer’s disease based on biomarker data [3, 4] the possibility to make a diagnosis of AD at the mild cognitive impairment (MCI) or predementia/prodromal– stage is becoming real, although currently biomarkers in asymptomatic or mild symptomatic persons should be used only in research settings as they are not yet validated for the use in the clinic [5, 6]. This requires additional care in the already difficult task of communicating an AD diagnosis avoiding to harm individuals [7]. Clear and loyal communication to the patient about the research character of the examinations and the uncertainty of the diagnosis are of paramount importance. As well as the communication of the degree and nature of the added value of the tests that refers to the potential incremented level of accuracy of the diagnosis, and to the consequent possible benefit of receiving results. These benefits may be more relevant in the case of prodromal AD, when the individuals themselves can personally take relevant decisions and plan for their future before and if cognitive deterioration occurs [8]. On the contrary, at present, a significant number of people never receive a diagnosis of dementia, and when diagnosis does occur, it is frequently late on in the disease process [9]. It follows that a large number of people at the first referral to specialist services are judged not to be competent to complete advance directives [10].

Cutting edge experimental treatments or other prevention trials are currently under way or in preparation [11]. Moreover, a recent study showed that a multi-domain intervention could maintain cognitive function and reduce cognitive decline in older at-risk individuals from the general population [12] thus providing promising results, which need to be further confirmed, on the possibility of cognitive impairment prevention. Anyway, at present, effective treatments for AD and other kind of dementia are not available. In this context, the possibility to plan for the future is one of the main advantages of an early diagnosis [13]. This possibility needs therefore to be a real option for subjects who receive diagnostic results and are still able to participate in decision-making processes; physicians should discuss the issue as soon as possible after a diagnosis is reached [14].

The legal status, if any, of advance directives and their regulation differs by country on the basis of the local socio-cultural context that sometime makes it difficult to fire a law. Anyway, advance directives are largely seen as an effective tool for planning medical care in the event the subject become incompetent. Their value has been nevertheless questioned with specific regard to people with dementia by scholars who refer to the arguments of personal identity and of patient’s changing interests before and after the onset of dementia.

In this paper, I discuss the value of advance directives in AD, taking into consideration major critics and providing reasons in favor of their use by patients at an early stage of the disease and their respect by medical doctors. The new possibility to use biomarkers for making a diagnosis of AD at the prodromal stage makes the ethical and legal discussion of the topic even more relevant.

## Main text

### Questioning the value of advance directives in dementia

#### The argument of personal identity

The value of advance directives in dementia has been questioned by scholars who refer to the argument of personal identity (see for instance: [15–17]). The argument of personal identity says that at the time patients with dementia become incompetent, they may be a different and a new person, while the earlier person (the person they were) is no longer in existence. In this situation, the advance directives issued by the earlier person cannot be applied to the person they become, for the basic reason that one person’s advance directives has no moral authority to determine treatment decision for another person. The argument assumes that psychological continuity is, as in Derek Parfit’s view [18], a necessary condition for personal identity over the time, and that patients with dementia may suffer such severe and permanent neurological damage that psychological continuity is so disrupted that they are no longer the same person.

The rich discussion on personal identity in patients with severe dementia testifies how interesting is this perspective from a theoretical point of view. However, it appears to have no ground in the real life where, on the contrary, it would have very bad consequences if taken seriously.

In the real life, patients fear to receive a diagnosis of dementia not as they could fear to know they are ending their existence; and they are worried about their own future with the disease not about another person’s future. Potential changes in personality, in believes and interests as consequence of the disease are perceived to be terrifying precisely because people feel that these changes will regard themselves and not that they will give rise to a different and new person. The same is true for the patients’ families and friends. The complaint that “Dad is no more dad” is just a sad metaphor that well represents how hard it is, as a family, to live with the disease. Patients with dementia, at whatever stage of the disease, are still recognized as mother and father, sister and brother, friend and fellow. Relatives and friends do feel to have responsibilities and duties towards them not just because they are part of the large human family, but because they are the very same person they used to be. Preservation of artistic and creative skills in AD patients until late in the disease course, despite progression in cognitive deficits, may also lay in favor of the personal identity continuity [19].

Beside being far from the patients and families’ real life, the discontinuity view would also have curious practical consequences. As Robert Olick has pointed out [20], the adoption of this thesis would necessitate substantial restructuring of important social, cultural and religious institutions, practices and values. In his discussion of the topic, he takes the identity argument to the extreme: if the earlier person is no longer in existence when severe dementia occurs, she may be defined as newly dead. This redefines the death of a person as a separate event from the death of the body, with the additional issues of what sort of grieving process may be possible in this circumstance, how family law could be modified to make clear that the dead patient’s family is not the family of the new person, how life and health insurance contracts could be revised to conform to the new definition of death, and so on.

Comparing to any psychological continuity standard, bodily identity, i.e. the assumption that bodily continuity is enough for personal identity, has huge advantages as a criterion of same-individual identity. Assuming bodily continuity corresponds to the real life of the persons and has practical favorable consequences for social and political practices. Furthermore and importantly, to look at the patient’s bodily continuity, and therefore to consider the person with AD the same person she was, better guarantees the respect for the person’s rights and wellbeing both before and after incompetence. In particular, it guarantees the incompetent person’s right to have a biographical history (even though she is not able any more to fully appreciate it), meaningful relationships (their meaning and value come from the past and are still recognize at least by relatives and friends), societal inclusion (that is maintained by the loved persons), and protection of personal interests (it would not be clear, otherwise, what kind of external entity should protect them).

#### The argument of conflicting interests

To share the view that the person with dementia is the same person she was before the disease does not solve the issue of the patient’s potential conflicting interests before and after severe dementia occurs. This has been described through case studies, as the famous case, reported by Firlik, of Mrs. Margo, who, despite Alzheimer’s disease, enjoys her life and appears to be a very happy person [21]. Suppose, as Dworkin did [22], that Margo when fully competent executed a formal document directing that, if she should develop AD, she should not receive treatment or even be killed in the event of any other serious disease she might contract. In this scenario of conflicting interests, someone may legitimately wonder if the patient’s actual interests should prevail on previous ones and advance directives should therefore be disregarded.

Dresser argues in favor of adopting a present best interest standard that requires systematic assessment of the existing interests of the individual incompetent patient [15]: in her view, it is a non sense to claim that matters such as dignity, privacy and bodily integrity (that are arguably integral to the well-being of the average or reasonable competent person in our culture) may affect the well-being of many incompetent patients with severely compromised mental abilities. Honoring the past treatment preferences could therefore inflict significant harm on the incompetent patient.

On the contrary, Dworkin describes Margo’s (and all other demented patients’) actual and previous interests as experiential vs critical interests. In his understanding, critical interests are those that give meaning and coherence to our life; they are second order interests and are much more important for the individual than experiential ones. In this view, advance directives, that protect individuals’ critical interests, should be honored.

Even though the claim that persons wish to live a coherent life and be consistent with their own values and believes until they die has been questioned [23], I believe that the execution of an advance directive is per se a proof that that claim is true for the individual who wrote the directive. Therefore, while there may be some reasons to give priority to experiential interests in the absence of a patient’s clear indication, the individual’s choice in favor of critical interests stated in an advance directive needs to be honored.

### Advance directives are especially advisable in dementia

Over the past years, significant efforts have been made to promote the principle of the respect for persons and their autonomy in the medical field. Despite the fact that there are some faults in their concrete implementation, advance directives are largely recognized to be an effective tool to promote subjects’ self-determination in the event the person becomes incompetent and actual informed consent is no longer possible. I believe that advance directives should be promoted for all citizens, to give voice to patients’ values and preferences (i.e. to patients’ view on themselves and life and on how to better promote and preserve that view) over the values and preferences of medical doctors or family members. Advance directives maintain a great moral and practical value even in countries where their legal value may be questionable. There are, however, additional reasons to support advance directives in the early stage of AD. In fact, in this case, the possibility of expressing care preferences in advance would be offered to the subject in a time where the disease is already detected, although symptoms are absent or very mild. This has the potential to overcome one of the major limit of advance directives, i.e. the fact that, when executed for broad future situations without specific cognition of what may happen to the person, they have an inevitable character of abstractness and potential ambiguity [24]. On the contrary, although the disease may appear in slightly different ways and time of progression in different patients, AD has a quite well known course that can be communicated to the patients, to enable them to take informed and enough circumstantial choices.

In addition, the directives would be eventually executed not too much in advance of future incapacity so that medical doctor should be able to present actual care and research options, as well as, realistically, the major lines of future research and possibilities. These elements are of great importance, although the gap of knowledge about how patients actually feel when they are severely demented [25] prevents the individuals executing the directive to take into consideration their future feelings.

Finally, far from being a reason to question the value of advance directives in patients with dementia, changes in personality and interests, as well as loss of memory, loss of the ability to reconstruct a biographical history, and loss of the ability to fully recognized family and friends, should be a further motivation for their promotion and concrete implementation. Those changes may in fact be perceived as worse than unconsciousness. Patients’ feelings on this regard need to be respected. The possibility to execute an advance directive has the potential to make people, who are worried about the effects of the disease progression on their mind, more confident of their decision power and less afraid about the future. This may also prevent extreme solutions, as planning suicide before one becomes demented [26], potentially loosing years of good life. Pre-emptive suicide would be even more problematic in the event the choice is based on the use of biomarkers that are still under research or that may generate just imperfect information about future disease [27].

### Which kind of advance directives for patients with dementia

According to national laws and local regulations, advance directives may contain both decisions regarding health care planning**,** including choices related to quality of life, and/or the appointment of an attorney in fact. This mean that people may indicate what medical treatment and care they do or do not want if, in the future, they are unable to make their wishes known, and/or appoint someone to make medical decisions for them if, in the future, they are unable to make those decisions for themselves. It is important that, along with decisions on ordinary medical treatment and care, the patient is invited to make decisions on particularly sensitive issues such as participation in research and end of life. A values history document could also be appended to an advance directive to help guide difficult care decisions and make them more reflective of the patient’s believes and priority [28].

#### Appointment of an attorney in fact

Advance directives that include both decisions regarding health care and the appointment of an attorney are particularly valuable and should be recommended [29]. They combine a certain amount of flexibility with the patient’s concrete instructions and seem to be the best possible way to give voice to AD people in the concrete situations of life. The attorney in fact should be a family member or a close friend whom primary concern is the patient’s well-being and whom the patient trust to make serious decisions. She/he should be a person who has spent time and shared experiences with the patient and is therefore able to give a voice to the patient’s wishes, values and believes. A trusted person should contribute to making decisions on the basis of the therapeutic indications given by the patient, and on the basis of his/her values and past life, but in the context of current medical/scientific possibilities and taking into consideration the peculiar situation of the patient. This will further guarantee that the already quite circumstantial advance directives executed at the beginning of the disease will be adapted in the best possible way to the very specific possibilities of care for the individual patient. The presence of an attorney in fact will also make easier for medical doctors, who have a limited or no knowledge of the patient, the task of interpreting directives in the case more than one intervention may be regarded as equal from a clinical perspective. From the other hand, knowing which treatment is consistent with the patient’s preferences may reduce the negative emotional burden that surrogates experience as the result of making treatment decisions for others [30].

Studies data seem to support a proxy appointment. Surrogate consent for dementia has broad support among the older general public [31, 32], and people seem to be willing to grant at least an amount of leeway to their surrogate [33]. Moreover, it is reasonable, although evidence is lacking, that data showing that surrogates’ judgements about patients’ preferences both in the clinical [34] and research setting [35] are often discordant could be corrected by prior written and not too broad communication of preferences by the patients themselves.

#### Participation in research

In the case of AD diagnosis at a prodromal or early stage, where patients anticipate cognitive decline and the loss of capacity and effective treatments are not yet available, an advance directive to prospectively consent to research participation is especially valuable. In this context, the participation in research protocols may be, under certain conditions, the best available option for them, and, in any case, research is the only way to eventually find a cure for the disease. The topic has been discussed both in US, where the National bioethics advisory committee recommended official recognition of such directives [36], and in Europe, where organizations promoting care for patients suffering from dementia [37] have supported the use of advance directives for research, provided that a number of safeguards are in place. Although, at least in the European regulations governing biomedical research, the legal status of advance directives for research is unclear [38], I regard as indisputable the moral value (that does not differ from the moral value of advance directives for medical treatment) and practical utility of this tool. The practical utility of advance directives for research is even higher than for medical treatment as the balance between benefit (if any) and risk is more difficult to estimate and is therefore harder for medical doctors and family members to take decision on behalf of the patient without a clear indication of preference. In this sense, an indication should be given in the advance directive also about the level of risk and burden that would be acceptable by the patient.

An advance directive for research could be implemented even in the absence of an attorney’s appointment [39]. Anyway, differently from advance directives for medical treatment, the presence of a caregiver who shares the patient’s choice is an essential requirement to make the directive working, at least for the majority of dementia clinical trials that require the availability of a caregiver as an inclusion criterion for research participation. In addition, while it is true that the medical doctor should be able to describe to the patient at an early stage of AD the major lines of future research, he will not be able to be as specific as in the presentation of ongoing research protocols. An attorney in fact would be of great help in applying the patient’s directive to the very specific research protocol and in deciding in favor or cons the participation in the study, according with the patient’s wishes. In any case, the use of an advance directive as a sign of willingness to participate in research is not a substitute for other requirements for the implementation of biomedical research involving patients without capacity. These requirements set out the conditions for offers of research participation to such patients and should be fulfilled. They require, among other conditions, that the research has the potential to produce real and direct benefit to the participant’s health or, where the research has not that potential, the research has the aim to contributing to the ultimate attainment of results capable of conferring benefit to the person concerned or to other persons having the same condition, and the research entails only minimal risk and minimal burden for the individual concerned [40]. Moreover, the right to withdrawal from research at any time should be granted to any participant: for the ones not able to change their mind (as in the case of participation in research based on an advance directive) others should take care of their well-being during the trial [41]. Ethics committees, whose role is to ‘protect the dignity, rights, safety and well-being of research participants’[40] play a crucial role in the evaluation of all those ethical requirements and in the guarantee that no one is treated as a mere object or mean of the experiment.

#### End of life decisions

End of life decisions are the most controversial issue concerning advance directives for patients with dementia as they usually aim to terminate a life that the patient, when competent, judged not worth to live. Anyway, advance directives in the case of dementia may be not only focused on refusal of treatment. A person should be equally supported and encouraged to express a wish to receive whatever form of appropriate medical treatment and/or care is available to prolong her life [42]. In this light, the society has a duty to ensure that people have a real choice and are not forced to renounce to treatments: the availability and quality of services and health care, including high quality palliative care should be granted for all patients with dementia.

Despite the availability of services and care and every effort to reassure the patient on future assistance, the person may nevertheless judge her future life with dementia unacceptable and may wish to terminate life refusing life-saving treatments, including artificial nutrition and hydration. Those refusals are widely established as a competent person’s right, and the physicians should fully respect the patient’s advanced dispositions on this regard, as they have to respect patients’ actual wishes on treatment refusal. The fundamental value options, including dignity of the human person, relational autonomy, quality of life and care [43], are important for an ethical evaluation of end of life decisions in persons with dementia precisely as in persons with other diseases*.* Not to honor a demented patient’s advance directive would be to treat the actually incompetent person as she had never been competent [44] and able to have her own view on herself and her life. On the contrary, one component of treating persons with respect is to view them as they view themselves, i.e., at least for people who executed an advance directive, as the unified subject of a human life that transcend the state of incapacity [45].

### Difficulties in the implementation of advance directives in dementia

People with AD need to be reassured that their advance directives will be implemented, just as the directives of people in other disease conditions. The circumstance that a person suffering from dementia will lack the capacity for independent decision making that would enable her to eventually revise her earlier choice in the case she will find dementia acceptable [46] just need to be well explained to the patients who may be not aware of it. By the way, the execution of an advance directive has precisely the meaning to avoid that interests other than the earlier competent person’s interests will prevail. Moreover, patient’s changing interests cannot be explained as a ‘response shift’ as it is very unlikely that patients with severe dementia change their internal standards, their values and their conceptualization of quality of life [47].

The unquestionable moral value of advance directives in dementia and the patient’s right to have his directive implemented do not solve every question regarding the patient’s treatment. In particular, the progressive worsening of the patient’s condition along a continuum between no or very mild symptoms to a complete state of dependency makes it difficult to identify the right point in time for enacting the patient’s directive to avoid both a too early and a too late implementation. Even the supporters of the arguments of changing personal identity or changing interests would accept to honor the patients’ advance directives in the case of individuals who retain bodily function but are no longer conscious, as in such situation there is no “new person” with “actual interests” to compete with the earlier person. Nevertheless, the implementation of an advance directive at this stage of the disease would probably be too late to respect the patient’s request. On the other side, a person with AD who clearly remembers her former self, values and believes does not give rise to doubts regarding the continuity of her personal identity. Moreover, this person should still be able to choose for herself (regardless of her legal status) and it should not be yet the time to turn to an advance directive. The more challenging situation is when the patient is neither able to choose nor unconscious, but in a conscious, severely demented and debilitated state with limited experiences. If, in addition, the person seems to appreciate life, the question of the social tolerability of following any kind of advance directive of a happily demented person is crucial [48]. Following an advance directive involves in fact the individual physician who participates and the medical community as a whole, as well as friends, family members and other caregivers who are part of the person relationship. It is therefore understandable that the values of the community play some role, as study results on physicians’ attitudes show [49]. I maintain that, if the person is not fully competent and there appears to be a conflict between current and former wishes, the person’s current wishes and feelings should be taken into consideration as they represent the person’s current mental and emotional state and attitudes [42]. Moreover, I agree on the importance of a case by case evaluation to carefully assess any relevant element of the situation at hand, including the precise characteristics of the advance directive [50].

Nevertheless, as I have argued, current interests should not override the indications of a clear and not too broad advance directive, and the values of others**,** including those of the attorney in fact, should not prevail over the patient’s preferences stated in the advance directive**,** as they do not prevail in the case of a competent person.

For a directive to be effective, problems that have been identified in the general population with advance directives implementation should also be overcome: they mainly consist in low health literacy, unclear use of terminology, lack of communication with the proxy, and advance directive accessibility [51]. An analysis of the content of demented patients’ advanced directive have also shown that they often convey conflicting wishes, or are improperly completed, or do not delineate explicitly the various treatments patients would or would not want, and do not address many common end-of-life care decisions [52]. Elderly people, including individuals at an early stage of AD, are likely to be not fully confident with the use of an advance directive, and directives of low medical quality can contribute to low compliance by the physicians [53]. Finally, advance directives cannot ask for clinically inappropriate health care or treatment or for anything that falls under the scientific community’s judgement of futility that applies to every medical intervention in any situation of life. The assumption here is that people cannot ask in their advance directives more than what can be asked to the medical system by a competent person.

## Conclusions

The prevalence of advance directives among old people, including patients with dementia, is quite low, and physicians rely on their medical judgement and family view more than on patient’s expressed preferences [53]. Few and not conclusive data exist on the potential positive impact of advanced directives on the treatment of patients with severe cognitive impairment or dementia, for instance regarding avoiding tube feeding or transfer from nursing home to hospital and receiving palliative care [54–56].

This requires concrete policies and actions to make directives known and effectives, particularly in a context of increased possibility of early diagnosis of AD paired with lack of effective treatments. Although different countries may have different regulations, the family physician could play a key role in encouraging the use of directives by people at an early stage of AD, in informing and educating about the correct execution of an advance directive, as well as in assuring its ongoing revision and its availability any time and in any context of cure [51].

To promote and honor advance directives in dementia is not in contrast with the acknowledgement of the full dignity of the person with dementia nor with the call to the personal and societal duty to care for the others. On the contrary, not to offer the patients with AD at a prodromal or early stage the opportunity to execute an advance directive or not to honor that directive would be an unacceptable form of discrimination towards demented patients.

## Abbreviations

AD: Alzheimer’s disease
MCI: Mild cognitive impairment
